# Hepatocellular carcinoma converted from atezolizumab and bevacizumab combination therapy: a case report

**DOI:** 10.1093/omcr/omae155

**Published:** 2024-12-28

**Authors:** Keisuke Ida, Satoshi Koizumi, Atsuhito Tsuchihashi, Shinjiro Kobayashi, Takehito Otsubo

**Affiliations:** Department of Gastroenterological and General Surgery, St. Marianna University School of Medicine, 2-16-1 Sugao Miyamae-ku, Kawasaki-shi, Kanagawa-ken 216-8511, Japan; Department of Gastroenterological and General Surgery, St. Marianna University School of Medicine, 2-16-1 Sugao Miyamae-ku, Kawasaki-shi, Kanagawa-ken 216-8511, Japan; Department of Gastroenterological and General Surgery, St. Marianna University School of Medicine, 2-16-1 Sugao Miyamae-ku, Kawasaki-shi, Kanagawa-ken 216-8511, Japan; Department of Gastroenterological and General Surgery, St. Marianna University School of Medicine, 2-16-1 Sugao Miyamae-ku, Kawasaki-shi, Kanagawa-ken 216-8511, Japan; Department of Gastroenterological and General Surgery, St. Marianna University School of Medicine, 2-16-1 Sugao Miyamae-ku, Kawasaki-shi, Kanagawa-ken 216-8511, Japan

**Keywords:** hepatocellular carcinoma, conversion surgery, targeted therapy

## Abstract

Hepatectomy is the curative treatment for hepatocellular carcinoma (HCC), with targeted therapy used when resection is difficult. In this rare case, the tumor shrank with targeted therapy, enabling radical treatment through conversion surgery. The patient, a man in his 70s, developed an 11.5-cm HCC (T3N0M0 Stage III) in the posterior hepatic zone after being virologically negative for hepatitis C. The tumor was near the right portal vein branch, making the liver lobe unresectable due to poor liver function. Upon tumor enlargement, he was treated with atezolizumab and bevacizumab. After 5 courses, the tumor significantly shrank, allowing for complete resection with posterior segment removal. The surgery revealed mostly necrotic tumors with no active cancer cells remaining.

## Introduction

In general, the curative treatment for hepatocellular carcinoma (HCC) is hepatectomy. Radical resection is possible if the tumor invades up to the first branch of the portal vein. Targeted therapy is used for HCC not only when the disease is advanced, but also when surgical resection and other local therapies are not indicated [1]. In recent years, guidelines have recommended effective regimens, such as the molecularly targeted agent lenvatinib [2] or the combination of an immune checkpoint inhibitor and a molecularly targeted agent, such as atezolizumab and bevacizumab [3]. Conversion surgery is increasingly being reported in HCC, in which radical resection is performed on lesions that are initially considered unresectable but have responded to drug therapy and become resectable.

## Case report

A 70 year old male patient with a medical history significant for hepatitis C, treated withinterferon therapy three years prior, and prostate enlargement. Despite persistent negative virological results on follow-up, the patient was referred to our department due to suspicion of hepatocellular carcinoma (HCC). Notably, the patient’s lifestyle includes daily consumption of 300–500 ml of whiskey with no history of smoking. Blood tests were negative for HBs antigen and positive for HCV antibodies. Liver function was Grade A with a Child-Pugh classification of 5 points, and liver damage was also Grade A. The ALBI score was −2.43, ALBI Grade 2a, and ICG R15 19.1%. Tumor markers were normal with CEA 2.5 ng/ml, but high AFP 1984 ng/ml and CA19-9 3135 U/ml.

**Figure 1:**
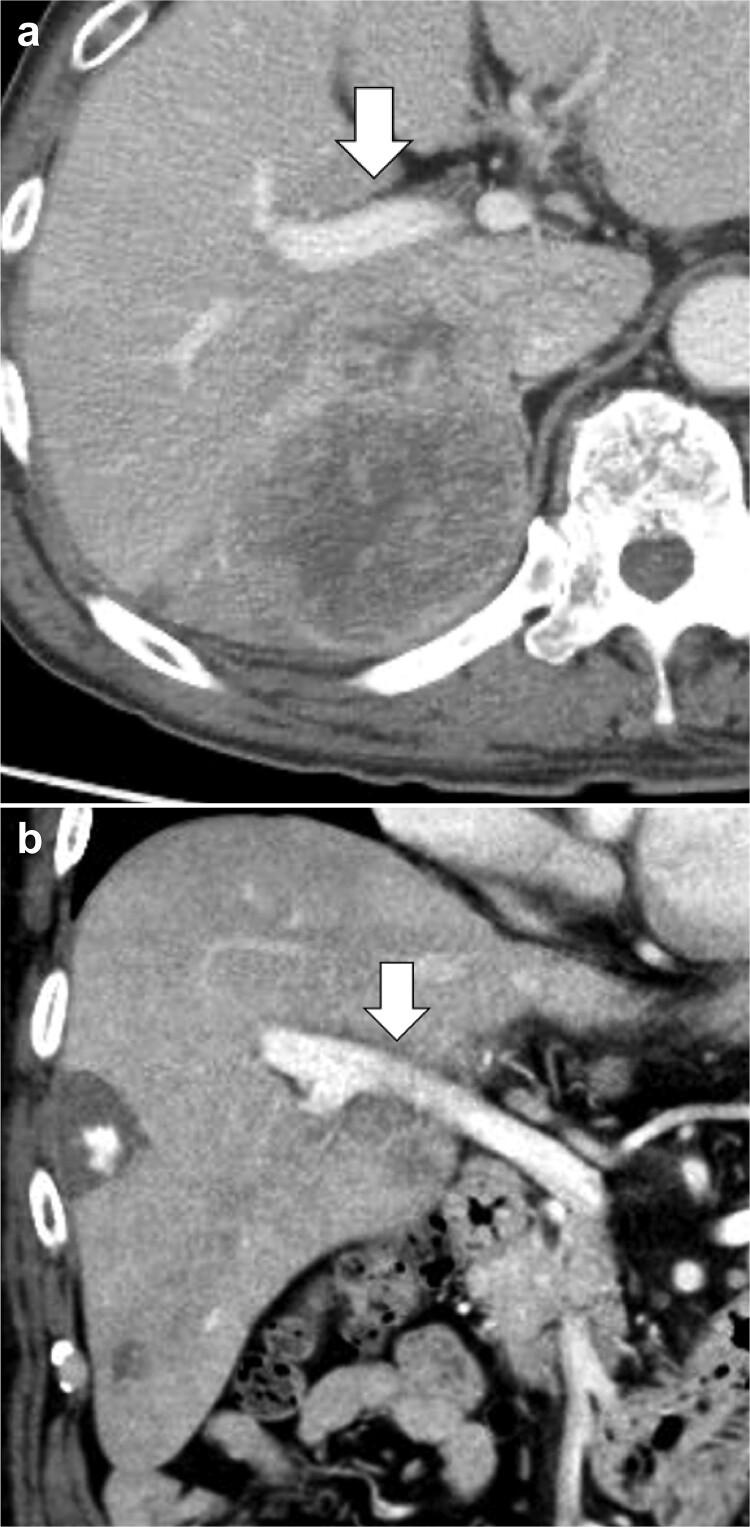
Initial abdominal contrast-enhanced CT scan. (**a**) A 9.1 × 9 cm large multinodular fused tumor was seen in the posterior region, predominantly in the caudal paravalvular region. HCC was diagnosed based on the contrast patterns. (**b**) The tumor was indistinctly bound to the first branch of the portal vein.

Contrast-enhanced computed tomography (CT) scan: A multinodular fused mass with a maximum length of 91 mm spanning the posterior segment and caudate lobe was observed. HCC was detected by dark staining in the early phase and by washout in the equilibrium phase. The primary branch boundary of the right portal vein was obscured (Fig. 1a and b). A hemangioma was seen in S5 of the liver, and a small cyst was observed in S6 of the liver.

Initial treatment strategy: The HCC diagnosis was cT3N0M0, cStage III. There was no border to the first branch of the portal vein, and right caudate lobe resection of the liver was considered a radical resection. However, the resection limit was 44% based on a residual liver ICG R_15_ of 40% [4], and resection was judged impossible because of poor residual liver function.

**Figure 2:**
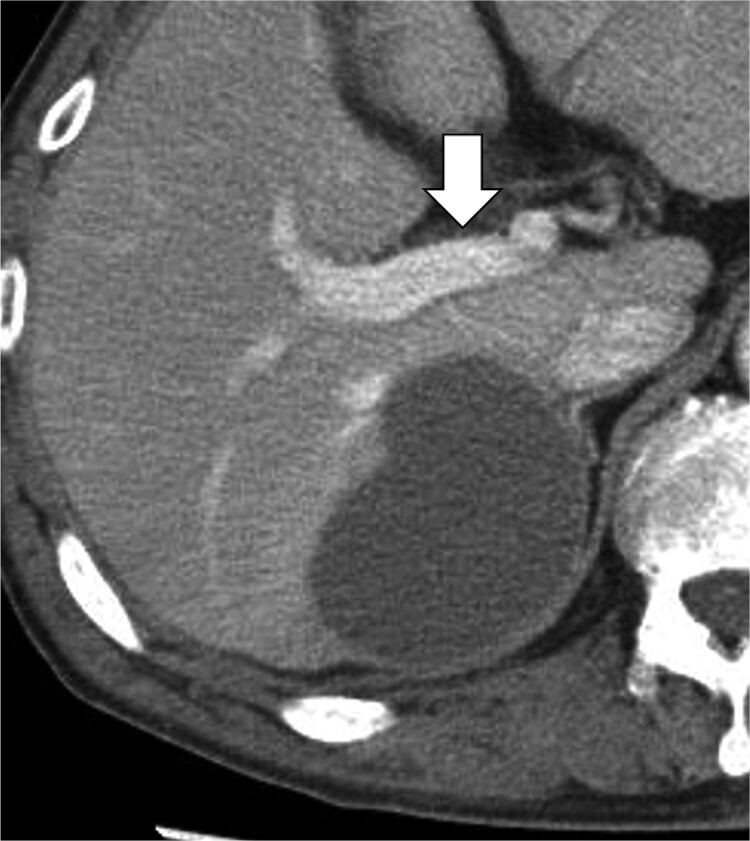
CT scan after drug therapy. The tumor shrank to a maximum diameter of 60 mm and the contrast effect disappeared. The tumor was a low-absorption mass. The tumor was located far from the first branch of the portal vein.

Targeted therapy: The initial treatment consisted of targeted therapy with a molecularly targeted agent (lenvatinib, 8 mg) and transcatheter arterial embolization (TAE). A CT scan performed 4 weeks after the start of treatment showed that the tumor had increased by approximately 120% to a maximum length of 110 mm. We switched to a combination of atezolizumab (1200 mg) and bevacizumab (1000 mg) targeted therapy, which has been reported to be useful in lenvatinib-negative cases [5]. No adverse events occurred with both targeted therapies. A CT scan performed after four courses of atezolizumab plus bevacizumab showed that the tumor had shrunk to a maximum diameter of 60 mm, the contrast effect had disappeared, and the mass had a generally low absorption (Fig. 2).

Tumor markers were normalized to 3.4 ng/ml AFP and 18 U/ml CA19-9.

Surgical findings: post-hepatic lobectomy. Blood loss was 293 ml. 6 h 23 min.

**Figure 3:**
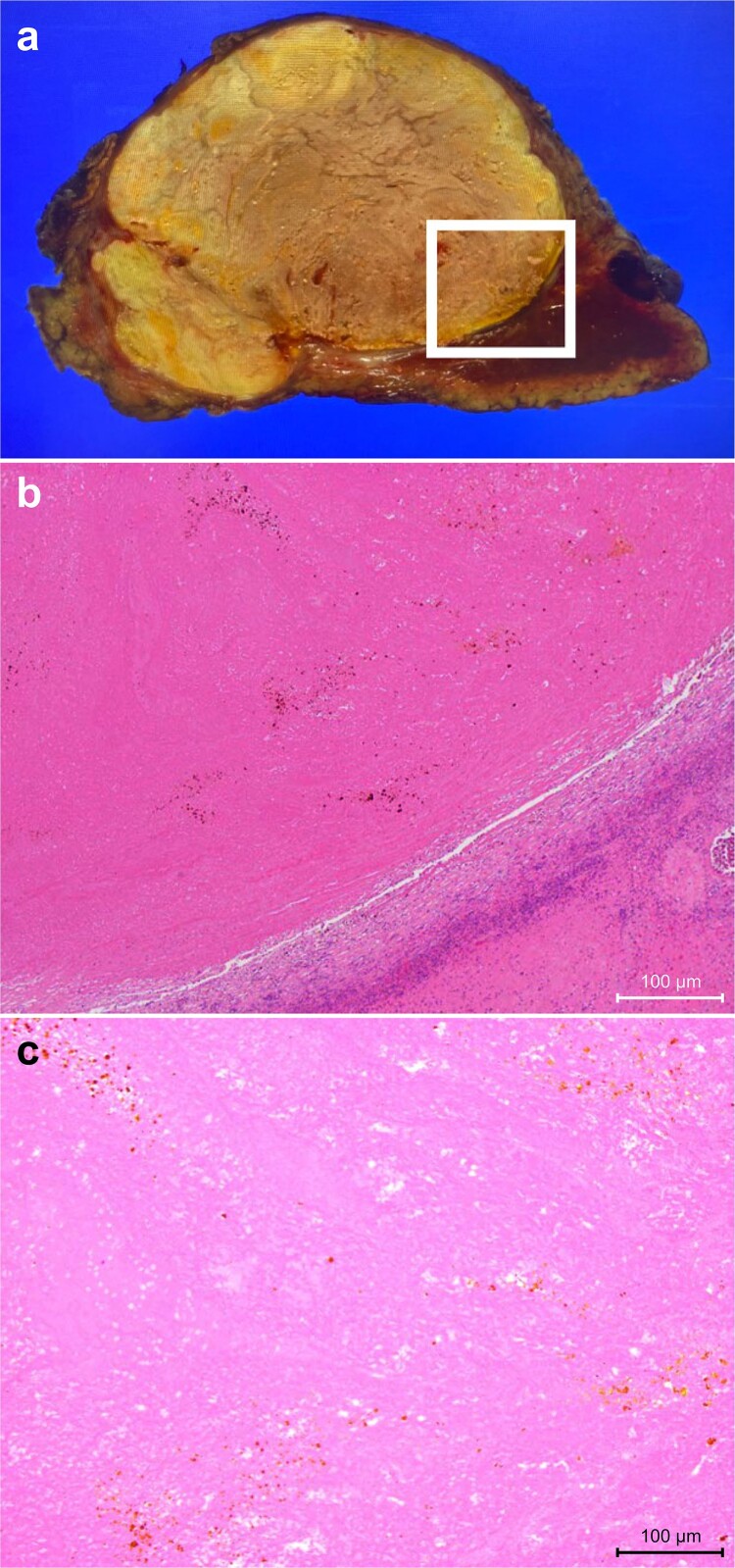
Pathological and histological findings. (**a**) Grossly, a yellowish-white tumor with other nodal fusion. (**b**) Weakly magnified image. Histologically, the tumor was almost completely necrotic, and epithelial granulomas were observed in the surrounding area, suggesting post-treatment changes. (**c**) Magnified image. The tumor was almost completely necrotic and no active cancer cells remained.

Histopathological findings: Grossly, the tumor was yellowish-white with other nodal fusions. Histologically, most tumors were necrotic, and there was no evidence of residual active HCC. Epithelioid granuloma formation was observed in the surrounding area, which was considered a post-treatment change (Fig. 3a–c).

Postoperative course: The patient showed no signs of postoperative liver failure; however, CT performed on postoperative day 7 revealed the formation of a truncal abscess. Percutaneous drainage quickly relieved the inflammatory, and the patient was discharged from the hospital on postoperative day 22 without drain removal. The drain was removed on postoperative day 38. The patient is currently alive 16 months postoperatively without recurrence. There is no recommended treatment after resection, and no additional treatment was provided.

## Discussion

In recent years, a wide variety of targeted therapy regimens have been developed for HCC, and favorable outcomes have been reported [6]. Many cases of unresectable advanced HCC have been reported in which the combination of lenvatinib and TAE resulted in tumor shrinkage and made conversion surgery possible [7]. The most frequently reported cases were those in which conversion surgery was performed after lenvatinib administration, and good results were reported when molecularly targeted therapy and TAE were combined with surgical therapy [8]. However, there have been few reports of conversion surgery after combination therapy with azolizumab and bevacizumab.

In the IMbrave150 study [3] that investigated the efficacy of the combination of atezolizumab and bevacizumab, the response rate was 27.3% and the complete response rate was 5.5%. On autopsy, the combination of atezolizumab and bevacizumab resulted in a complete response with no residual tumor on histopathological examination. The procedure itself was reduced because the tumor was removed from the primary branch of the portal vein. A similar case report of conversion surgery with a pathologically proven complete response [9] also suggests that this may be a useful regimen when conversion surgery is planned.

However, there have been reports of immunological adverse events during conversion surgery after atezolizumab plus bevacizumab combination therapy [10]; therefore, its safety has not yet been established. In our study, to avoid delayed wound healing with bevacizumab, it was omitted from the last of the five courses, and surgery was performed after an interval of four cycles. Although the causal relationship is unclear, the patient had some difficulty in controlling the infection, evidenced by the formation of an edge abscess. The RACB study [11], a clinical study on the safety and efficacy of a combination of atezolizumab and bevacizumab and multimodality treatment using surgical resection for unresectable HCC, is currently underway, and we are awaiting further results.

The results of this study suggest that conversion surgery after atezolizumab plus bevacizumab combination therapy may be a useful treatment option for HCC.
